# A Novel Organizational Pattern of the Nasofrontal Complex in *Plecotus auritus* Revealed by Micro-Computed Tomography

**DOI:** 10.3390/ani16162460

**Published:** 2026-08-07

**Authors:** Grzegorz Kłys, Paweł Socha

**Affiliations:** 1 Institute of Biology, University of Opole, ul. Oleska 22, 45-052 Opole, Poland; 2 Department of Palaeozoology, Zoological Institute, University of Wroclaw, ul. Sienkiewicza 21, 50-335 Wrocław, Poland; pawel.socha@uwr.edu.pl

**Keywords:** Chiroptera, *Plecotus auritus*, cranial anatomy, micro-computed tomography, comparative anatomy, nasofrontal complex

## Abstract

The nasofrontal region of mammals is generally characterized by direct contact between the paired nasal bones and the frontal bone. However, detailed spatial relationships among the frontal bone, nasal bones, and osseous nasal septum remain insufficiently documented in bats. This study investigated the three-dimensional organization of the nasofrontal complex in the brown long-eared bat (*Plecotus auritus*) using high-resolution micro-computed tomography. Ten adult skulls were analyzed using three-dimensional reconstructions and multiplanar image analysis. A consistent and previously undescribed organizational pattern was identified in all specimens. Its principal feature was a median rostral extension of the frontal bone, designated *processus internasalis ossis frontalis* (nova structura), which projected between and separated the caudal portions of the paired nasal bones. This structure was consistently associated with *ala ossis nasalis* (nova structura), *facies parasagittalis ossis nasalis* (nova structura), a pronounced internasal sagittal impression, and a close topographical relationship with the osseous nasal septum. Together, these features constitute an integrated morphological complex rather than isolated anatomical modifications. The observed arrangement differs from the generalized mammalian condition, in which the paired nasal bones maintain direct median contact at the nasofrontal boundary. These findings reveal a distinct pattern of nasofrontal organization in *P. auritus* and demonstrate the value of high-resolution micro-computed tomography for identifying previously unrecognized aspects of mammalian cranial anatomy.

## Simple Summary

The bones of the mammalian nasal region are generally thought to follow a common anatomical pattern. Using high-resolution micro-computed tomography, we examined the skull of the brown long-eared bat (*Plecotus auritus*) and discovered a previously undescribed organization of the nasofrontal complex. We identified a new bony projection of the frontal bone extending between the paired nasal bones together with additional associated anatomical structures. These features were consistently present in all examined specimens and show that the nasal region is organized differently from the generalized mammalian pattern. Our findings demonstrate that high-resolution micro-computed tomography can reveal anatomical features that have previously remained unrecognized, providing new insights into the comparative anatomy and evolution of the mammalian skull.

## 1. Introduction

The nasofrontal complex represents one of the principal regions of morphological integration within the mammalian skull, linking elements of the viscerocranium, neurocranium, and the osseous framework of the nasal cavity into a unified structural and developmental system [1,2,3]. It is composed primarily of the frontal bone (*os frontale*), the paired nasal bones (*ossa nasalia*), and the deeper components of the nasal skeleton, including the osseous nasal septum (*septum nasi osseum*) and the ethmoid bone (*os ethmoidale*). The spatial relationships among these structures determine the architecture of the dorsal skull and the organization of the anterior nasal cavity, contributing to the protection of sensory organs as well as to the formation of the respiratory and olfactory systems [1,3].

Despite the conserved general organization of the mammalian skull, the nasofrontal region exhibits considerable evolutionary variation associated with adaptations to diverse environmental conditions, feeding strategies, sensory specializations, and biomechanical modifications of the cranium [2,4]. Comparative studies have focused primarily on variation in the shape and proportions of individual cranial bones, their homologies, and the configuration of cranial sutures, whereas the three-dimensional organization of the nasofrontal complex has received comparatively little attention; the frontal bone is defined by a direct articulation through the nasofrontal suture (sutura frontonasalis), which is regarded as the typical organizational pattern in mammals [1,3]. However, the detailed three-dimensional architecture of this region remains relatively poorly understood, as most previous investigations have relied on the examination of external bone surfaces or conventional anatomical preparations that do not permit comprehensive reconstruction of the spatial relationships among structures concealed within the skull.

Over the past two decades, the development of high-resolution micro-computed tomography (micro-CT) has fundamentally advanced comparative anatomical research in vertebrates by enabling non-destructive visualization of skeletal structures at a resolution sufficient to examine even the finest osseous details and the course of cranial sutures. Three-dimensional reconstructions generated from micro-CT datasets allow detailed assessment of spatial relationships among skeletal elements without the need for specimen preparation or destructive manipulation. Consequently, even well-studied regions of the skull may reveal previously unrecognized aspects of their anatomical organization [1,3].

Among mammals, bats (Chiroptera) represent a particularly informative group for investigations using high-resolution micro-computed tomography, as their skulls combine a high degree of functional specialization with small size and exceptionally complex three-dimensional organization.

Bats (Chiroptera) are among the best-studied mammalian groups with respect to cranial morphology, largely because of their remarkable adaptations for powered flight, echolocation, and the extraordinary diversity of their feeding strategies. Over the past several decades, numerous studies have investigated the osteology, ontogeny, biomechanics, and evolution of the chiropteran skull, substantially expanding our understanding of its structural organization and functional diversity [5,6,7].

The most comprehensive osteological studies have focused on the description of individual cranial bones, the configuration of cranial sutures, and their homologies. Among these, the detailed osteological account of the skull of Pteropus provided by Giannini et al. [5] remains one of the principal references for chiropteran cranial anatomy. Their work described, among other structures, the nasal bones, the frontal bone, and the nasofrontal suture (*sutura frontonasalis*), thereby establishing an important foundation for the modern osteological terminology of Chiroptera. However, these investigations were based primarily on classical descriptive osteology and did not include three-dimensional reconstruction of the spatial relationships among the elements forming the nasofrontal complex.

Despite substantial advances in studies of bat cranial anatomy and evolution, the three-dimensional organization of the nasofrontal complex remains insufficiently understood. Previous investigations have focused primarily on the morphology of individual cranial bones, the configuration of cranial sutures, embryonic development, biomechanics, and phylogenetic relationships [5,6,7,8,9]. In contrast, considerably less attention has been devoted to the spatial relationships among the frontal bone, the paired nasal bones, the osseous nasal septum, and the ethmoid bone as components of an integrated anatomical system. Consequently, it remains unclear whether the classical model of direct contact between the nasal and frontal bones adequately reflects the full range of morphological organization of the nasofrontal region in bats.

The classical anatomical model describes the relationship between the nasal bones and the frontal bone as a direct articulation through the nasofrontal suture (*sutura frontonasalis*), a concept derived primarily from the examination of external bone surfaces and conventional osteological observations [1,3]. The advent of high-resolution micro-computed tomography has transformed the study of cranial morphology by enabling non-destructive visualization of the internal architecture of the skull at a resolution sufficient to identify subtle spatial relationships among skeletal elements that may remain undetectable using traditional anatomical approaches [10,11].

Consequently, it remains uncertain whether the organization of the nasofrontal complex in bats conforms to the classical mammalian model or whether it encompasses previously unrecognized structural variants. Detailed investigations of the three-dimensional topography of the relationships among the frontal bone, the paired nasal bones, the osseous nasal septum, and the ethmoid bone in adult individuals are still lacking. Addressing this knowledge gap is important not only for the comparative anatomy of Chiroptera but also for improving our understanding of the evolutionary diversity of anterior cranial organization across mammals.

The selection of *P. auritus* as the study species was not incidental. This species is among the best-studied European bats in terms of its biology and has been the subject of numerous investigations addressing its ecology, foraging behaviour, echolocation, and functional cranial morphology. Despite this extensive body of literature, the detailed three-dimensional organization of the nasofrontal complex has remained virtually undescribed, providing a strong rationale for the present study.

*P. auritus* (Linnaeus, 1758), a member of the family Vespertilionidae, is one of the best-studied European bat species with respect to its biology, ecology, and general morphology. Nevertheless, despite this extensive body of knowledge, the detailed three-dimensional anatomy of its cranial skeleton remains insufficiently explored. The species has a broad geographic distribution, exhibits relatively low intraspecific morphological variation, and possesses well-documented reproductive biology, hibernation behaviour, and roosting ecology. Consequently, it has long served as an important study species in ecological and physiological investigations of bats [12,13].

From a comparative anatomical perspective, *P. auritus* is characterized by an elongated facial region of the skull, a well-developed nasal region, and distinctive proportions of the frontal and nasal bones. These features are closely associated with its highly specialized auditory system, exceptionally large pinnae, and a low-intensity echolocation strategy used during active prey detection on vegetation surfaces (gleaning) [13,14,15].

At the same time, the detailed osteology of the anterior region of the skull in *P. auritus* remains considerably less well understood than the species’ biology, ecology, and behaviour. Previous studies have focused predominantly on its ecology, behaviour, echolocation, and general cranial morphology, whereas the organization of the nasofrontal complex has not been investigated as a distinct anatomical entity. Consequently, *P. auritus* represents a particularly suitable study species for high-resolution micro-computed tomography, which enables detailed evaluation of the spatial relationships among the skeletal elements of the nasal region and the anterior neurocranium.

The present study represents the next stage of our systematic investigations into the cranial morphology of *P. auritus*. These studies have already revealed previously undescribed osseous structures and demonstrated the value of high-resolution micro-computed tomography for comparative anatomical research in this species [16].

An additional reason for selecting *P. auritus* was the availability of a substantial museum collection of specimens with well-documented provenance, enabling comparative analyses without disturbance to natural populations. The excellent preservation of the skeletal material, combined with imaging at micrometre-scale resolution, provided exceptional conditions for identifying subtle anatomical features that may remain undetectable using conventional osteological approaches.

The rapid development of high-resolution micro-computed tomography (micro-CT) over the past two decades has profoundly expanded the possibilities of comparative anatomical research by enabling non-destructive visualization of skeletal structures at micrometre-scale resolution [10,11]. Unlike conventional osteological approaches, which rely primarily on the examination of external bone surfaces and observations from a limited number of viewing angles, micro-CT provides comprehensive three-dimensional reconstructions of the skull, allowing detailed assessment of the spatial relationships among all skeletal components.

This approach is particularly valuable for studies of small mammals, in which many skeletal structures measure only a few to several tens of micrometres. Under such conditions, cranial sutures, delicate processes, thin osseous laminae, and contact surfaces between adjacent bones may remain partially or entirely undetectable using conventional osteological techniques, especially when located within the skull or obscured by surrounding skeletal elements [10,17].

The nasofrontal region represents one of the most structurally complex areas of the mammalian skull. It comprises thin osseous laminae, an intricate network of cranial sutures, elements of the ethmoid bone, and the osseous nasal septum, forming a highly complex three-dimensional anatomical arrangement. Examination of external bone surfaces alone is insufficient to reconstruct the true boundaries between individual bones or to determine their spatial relationships. Only three-dimensional reconstructions enable these connections to be traced in any plane and their continuity to be evaluated throughout the entire volume of the investigated region [11].

Accordingly, the application of micro-computed tomography in the present study was not merely an alternative imaging technique but an essential analytical approach for evaluating the classical model of nasofrontal complex organization. Three-dimensional reconstructions made it possible to trace osseous boundaries, examine the spatial relationships among skeletal elements, and identify anatomical features that would have been difficult to recognize using conventional osteological methods or would have required destructive specimen preparation. In this context, micro-CT served not simply as a documentation tool but as the principal method of morphological analysis, providing a level of anatomical resolution unattainable with traditional osteological techniques [10,11].

Despite substantial advances in mammalian comparative anatomy and the extensive body of morphological research on bats, the three-dimensional organization of the nasofrontal complex remains insufficiently understood. Although previous studies have provided detailed information on cranial osteology, embryonic development, biomechanics, and functional morphology [1,5,6,7,8,9], the spatial relationships among the frontal bone, the paired nasal bones, the osseous nasal septum, and the ethmoid bone have not been investigated as an integrated anatomical system. Consequently, it remains unclear whether the classical model of direct contact between the nasal and frontal bones adequately reflects the organization of the nasofrontal complex in mammals and bats or whether high-resolution three-dimensional imaging reveals previously unrecognized structural patterns. Addressing this question is important not only for the comparative anatomy of Chiroptera but also for a broader understanding of the evolutionary diversity of anterior cranial organization in mammals.

The aim of the present study was to test whether the classical model of nasofrontal complex organization, which assumes direct articulation between the paired nasal bones and the frontal bone, is supported in *Plecotus auritus* using high-resolution three-dimensional micro-computed tomography (micro-CT). A further objective was to characterize the spatial relationships among the skeletal elements forming this region and to identify anatomical features that have not previously been described. Together, these analyses provide a framework for reassessing the structural organization of the mammalian nasofrontal complex using high-resolution three-dimensional imaging.

## 2. Materials and Methods

### 2.1. Material

The study was based on the examination of ten adult skulls of *Plecotus auritus* (Linnaeus, 1758) obtained from the reference collections of the University of Opole and the Institute of Systematics and Evolution of Animals, Polish Academy of Sciences (ISEZ PAS), Kraków, Poland. All specimens consisted of dry museum skulls in good osteological condition and were suitable for micro-computed tomography (micro-CT), multiplanar image analysis, and three-dimensional reconstruction of cranial structures.

The analyzed material comprised the following specimens:

1.  PAR 15, 11, 2024. Stobrawski Landscape Park. Collection of the Institute of Biology, University of Opole-1_CZ;
2.  PAR Opole. Collection of the Institute of Biology, University of Opole 2_CZ;
3.  PAR 05, 09, 2023. Tarnowskie Góry. Collection of the Institute of Biology, University of Opole (no. 3-CZ);
4.  ISEZ PAS, specimen no. M/III90;
5.  ISEZ PAS, collected on 17 February 1972, Złoty Stok, Poland;
6.  ISEZ PAS, specimen no. M/11194, Solna Jama Cave, Gniewoszów, Poland;
7.  ISEZ PAS, collected on 5 April 1963, Zbójecka Cave, Ojców, Poland;
8.  ISEZ PAS, specimen no. M/1631/60, Hungary;
9.  ISEZ PAS, specimen no. M/11239/60, Radochowska Cave near Lądek-Zdrój, Poland;
10.  ISEZ PAS, specimen no. M/2450/62, Bielany, Kraków, Poland.

Only adult specimens were included in the study, as indicated by complete dentition and full ossification of the cranial elements. Juvenile specimens were excluded. Information on sex was unavailable for some specimens and was therefore not included in the analysis. Only complete skulls without visible mechanical damage in the region of interest were selected for examination.

### 2.2. Micro-Computed Tomography Scanning and Reconstruction

Micro-computed tomography (micro-CT) scanning was performed at the Laboratory of Micro-Computed Tomography, University of Wrocław, using a SkyScan 1273 system (Bruker, Kontich, Belgium). Acquisition parameters were optimized for the visualization of fine cranial skeletal structures and included an X-ray source voltage of 36–41 kV, a current of 111 μA, an exposure time of 2360 ms, a rotation range of 180°, a rotation step of 0.4°, and frame averaging of 10. The total scanning time per specimen was approximately 2 h 6 min. Reconstructed datasets had an isotropic voxel size of 9.2 μm and an image matrix of 3072 × 1944 pixels. No beam filter was used during scanning.

During image acquisition and reconstruction, flat-field correction and ring artefact reduction were applied. The scanning and reconstruction parameters were optimized to maximize image contrast and spatial resolution of fine skeletal structures, with particular emphasis on the nasofrontal region.

Image reconstruction was performed using NRecon software (version 2; Bruker, Kontich, Belgium). Standard reconstruction procedures included beam-hardening correction, signal filtering, and ring artefact reduction. Reconstruction and data-processing procedures followed the protocol used in a previous micro-CT study of the skull of *P. auritus* [16].

Reconstruction quality was assessed by visual inspection of cross-sectional images and three-dimensional reconstructions. Particular attention was paid to the preservation of fine anatomical details, the continuity of thin osseous structures, and the presence of potential imaging artefacts. Minor surface irregularities and localized noise observed in selected datasets were interpreted as technical artefacts and did not affect the identification of the principal anatomical structures or the assessment of their spatial relationships.

The reconstructed datasets were exported as 16-bit TIFF image stacks, preserving the full intensity range and original image geometry.

### 2.3. Digital Processing, Visualization, and Morphological Analysis

The reconstructed micro-CT datasets were processed to generate transverse, sagittal, and coronal sections for detailed examination of internal cranial structures. Three-dimensional reconstructions and segmentation of selected skeletal elements were performed to assess their spatial organization and anatomical relationships.

Data visualization, segmentation, and figure preparation were carried out using Dragonfly (version 2024.1; Object Research Systems, Montreal, QC, Canada) and Avizo (version 2023.2; Thermo Fisher Scientific, Waltham, MA, USA).

Morphological analysis was based on the integrated evaluation of cross-sectional images and three-dimensional reconstructions. Particular attention was devoted to the spatial relationships among the paired nasal bones, frontal bone, osseous nasal septum, and adjacent elements of the nasofrontal region. The continuity of osseous structures, the course of sutures, and the morphology of contact surfaces between adjacent skeletal elements were assessed.

Anatomical interpretations were based on repeated occurrence across specimens, continuity across successive image sections, and traceability within three-dimensional reconstructions. A morphological feature was considered anatomically valid only when it could be confirmed in both sectional images and three-dimensional reconstructions.

Figures were prepared from sectional data and three-dimensional reconstructions using consistent anatomical orientation, scale, and labelling conventions throughout the study.

### 2.4. Anatomical Terminology

Anatomical terminology followed the *Nomina Anatomica Veterinaria* [18], standard conventions used in mammalian and chiropteran osteology, and the terminological framework previously adopted for newly recognized cranial structures in *P. auritus* [16]. Classical anatomical structures were designated according to the established Latin anatomical nomenclature.

Morphological structures for which no corresponding terms were identified in the available anatomical nomenclature or previous osteological descriptions of Chiroptera were provisionally designated as *novae structurae*. In the present study, these include *processus internasalis ossis frontalis*, *ala ossis nasalis*, and *facies parasagittalis ossis nasalis*. These names are proposed as descriptive anatomical terms reflecting the morphology, topography, spatial relationships, and skeletal associations of the respective structures. The designation *nova structura* is used to indicate newly recognized morphological units and does not imply their formal inclusion in the official anatomical nomenclature.

To ensure terminological consistency and to distinguish official Latin anatomical terms from their English descriptive equivalents, all Latin anatomical terms and scientific names of taxa are presented in italics throughout the manuscript.

## 3. Results

The present study revealed a previously undescribed organizational pattern of the nasofrontal complex in *Plecotus auritus*, involving both newly recognized morphological structures (*novae structurae*) and modified spatial relationships among skeletal elements of the nasal region.

The principal observations included:

1. the presence of processus internasalis ossis frontalis (nova structura);
2. the occurrence of the internasal sagittal impression and lateral separation of the nasal bones;
3. the development of ala ossis nasalis (nova structura);
4. the presence of facies parasagittalis ossis nasalis (nova structura);
5. reorganization of the nasofrontal articulation;
6. a close spatial relationship between these features and the osseous nasal septum (septum nasi osseum).

### 3.1. General Morphology of the Nasal Bones

**Figure 1:**
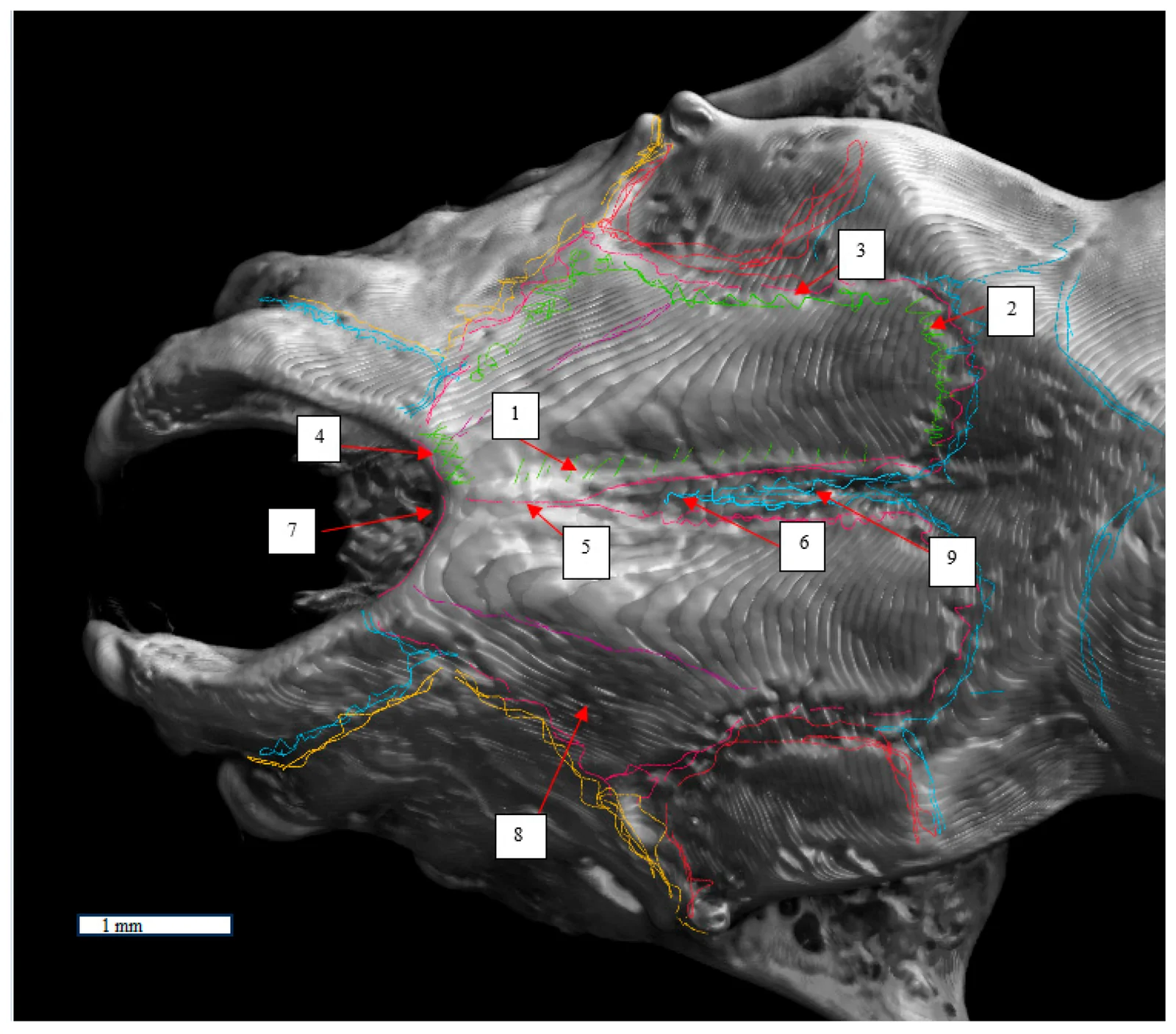
Dorsal view of the nasofrontal complex in *Plecotus auritus* (specimen no. 3). Paired nasal bones (*ossa nasalia*) exhibit a pronounced median organization associated with the course of the internasal suture (*sutura internasalis*) and a longitudinal sagittal depression designated as the *internasal sagittal impression*. The lateral margin of each nasal bone expands laterally into a broad laminar structure termed *ala ossis nasalis* (*nova structura*). In the caudal part of the nasal region, a rostral extension of the frontal bone forms *processus internasalis ossis frontalis* (*nova structura*), which projects between the paired nasal bones. The inferior margins of the paired nasal bones form the dorsal boundary of the piriform aperture (*apertura piriformis*). Colored lines delineate the boundaries of the anatomical structures shown. Scale bar = 1 mm. Labels: 1. Margo medialis; 2. Margo superior; 3. Margo lateralis; 4. Margo inferior (margo liber); 5. Sutura internasalis; 6. Internasal sagittal impression; 7. Vertex processus septalis; 8. Ala ossis nasalis (nova structura); 9. Processus internasalis ossis frontalis (nova structura).

The nasal bones (*ossa nasalia*) of *P. auritus* are paired, elongated elements forming the dorsal roof of the nasal region (Figure 1). In dorsal view, the nasal bones exhibit a pronounced median organization associated with the course of the internasal suture (*sutura internasalis*; Figure 1, label 5).

Along the midline of the paired nasal bones, a longitudinal sagittal depression, designated here as the internasal sagittal impression (Figure 1, label 6), extends parallel to the *sutura internasalis*. This structure divides the dorsal surface of the nasal region into two approximately symmetrical halves and was consistently observed in all examined specimens.

Each nasal bone exhibits a well-defined medial margin (*margo medialis*; Figure 1, label 1), superior margin (*margo superior*; Figure 1, label 2), lateral margin (*margo lateralis*; Figure 1, label 3), and free inferior margin (*margo inferior*, *margo liber*; Figure 1, label 4). The lateral margin is expanded and gives rise to a broad laminar extension designated here as *ala ossis nasalis* (*nova structura*; Figure 1, label 8).

The *ala ossis nasalis* forms an extensive lateral expansion of the nasal bone, increasing the width of the nasal roof, particularly in its median and caudal portions. In dorsal view, this structure gives the paired nasal bones a characteristically broadened outline.

Rostrally, the inferior margins of the paired nasal bones contribute to the dorsal boundary of the piriform aperture (*apertura piriformis*). Caudally, the paired nasal bones are separated by a median frontal-derived element designated as *processus internasalis ossis frontalis* (*nova structura*; Figure 1, label 9). This arrangement results in separation of the caudal portions of the nasal bones.

### 3.2. Ala Ossis Nasalis and Facies Parasagittalis Ossis Nasalis

**Figure 2:**
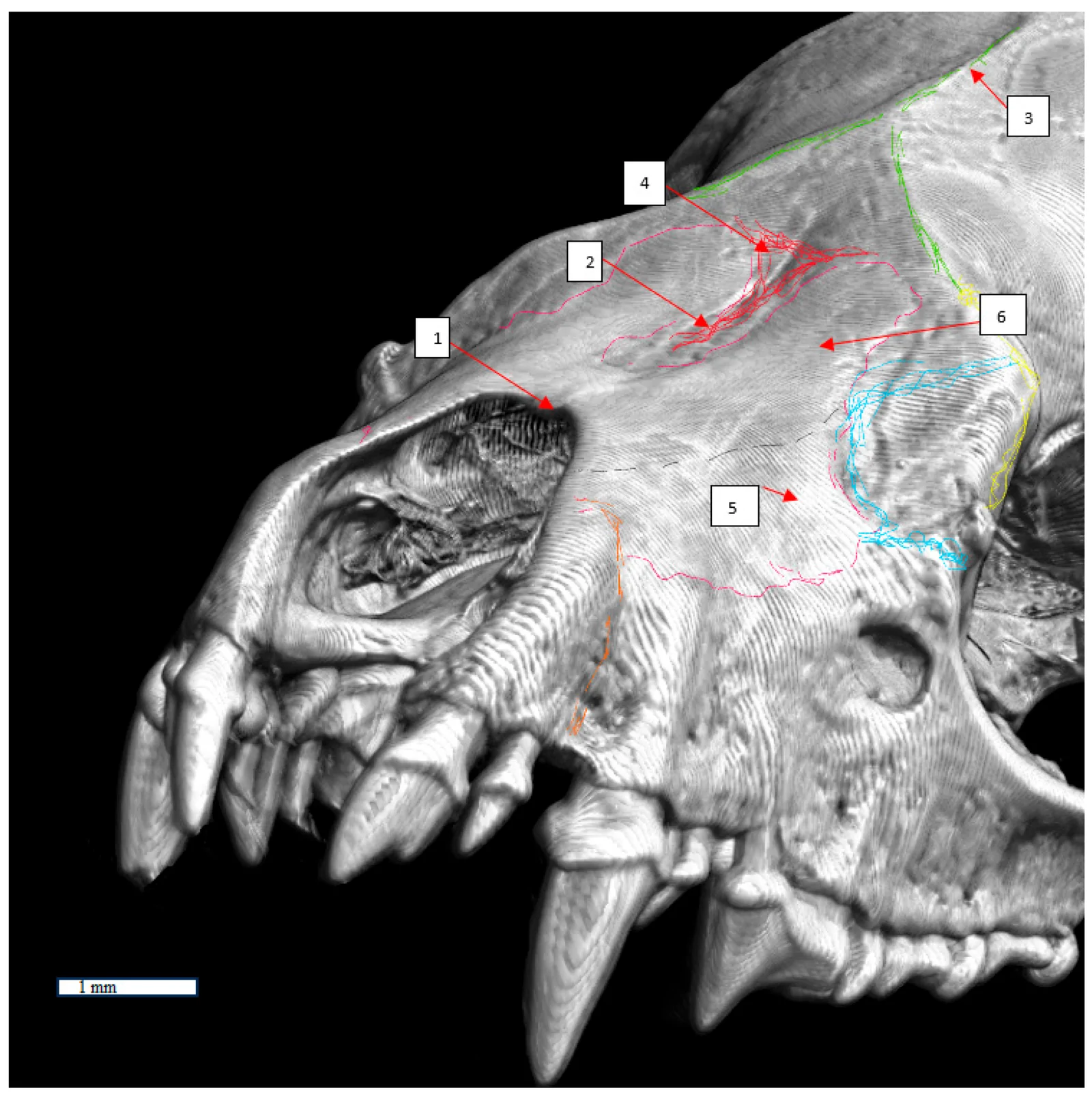
Oblique dorsolateral view of the nasofrontal complex in *Plecotus auritus* (specimen no. 3). This figure illustrates the spatial relationships among the nasal bones and the newly recognized anatomical structures associated with the components of the nasofrontal complex. Visible structures include the apex of the median complex (*vertex processus septalis*), the longitudinal sagittal depression designated as the *internasal sagittal impression*, the frontal crest (*crista frontalis*), the median frontal process (*processus internasalis ossis frontalis*; *nova structura*), the lateral expansion of the nasal bone (*ala ossis nasalis*; *nova structura*), and the parasagittal surface of the nasal bone (*facies parasagittalis ossis nasalis*; *nova structura*). Together, these structures characterize the median organization of the nasal region and its relationship to the nasofrontal complex. Scale bar = 1 mm. Labels: 1. Vertex processus septalis; 2. Internasal sagittal impression; 3. Crista frontalis; 4. Processus internasalis ossis frontalis (nova structura); 5. Ala ossis nasalis (nova structura); 6. Facies parasagittalis ossis nasalis (nova structura).

Along the lateral aspect of each nasal bone, an extensive osseous expansion is present and is designated here as *ala ossis nasalis* (*nova structura*; Figure 1, label 8; Figure 2, label 5). This structure forms a broad laminar plate extending laterally from the main body of the nasal bone. In dorsal view, it contributes substantially to the widening of the nasal region, particularly in its median and caudal portions (Figure 1).

Three-dimensional reconstruction demonstrated that *ala ossis nasalis* possesses distinct dorsal and lateral surfaces. In an oblique dorsolateral view (Figure 2), it forms an extensive superficial element occupying a considerable portion of the lateral aspect of the nasal region.

Medial to *ala ossis nasalis*, an elongated surface is present and is designated here as *facies parasagittalis ossis nasalis* (*nova structura*; Figure 2, label 6). This surface extends parallel to the long axis of the nasal bone and is situated between the median portion of the nasal roof and the lateral osseous expansion.

*Facies parasagittalis ossis nasalis* remains in direct topographical continuity with the internasal sagittal impression (Figure 1, label 6; Figure 2, label 2). In an oblique dorsolateral view, these structures are clearly distinguishable and form separate surface components of the dorsal nasal region.

All examined specimens exhibited both *ala ossis nasalis* and *facies parasagittalis ossis nasalis*. Their morphology, position, and relationships with adjacent structures were consistent throughout the analyzed material.

### 3.3. Processus Internasalis Ossis Frontalis

**Figure 3:**
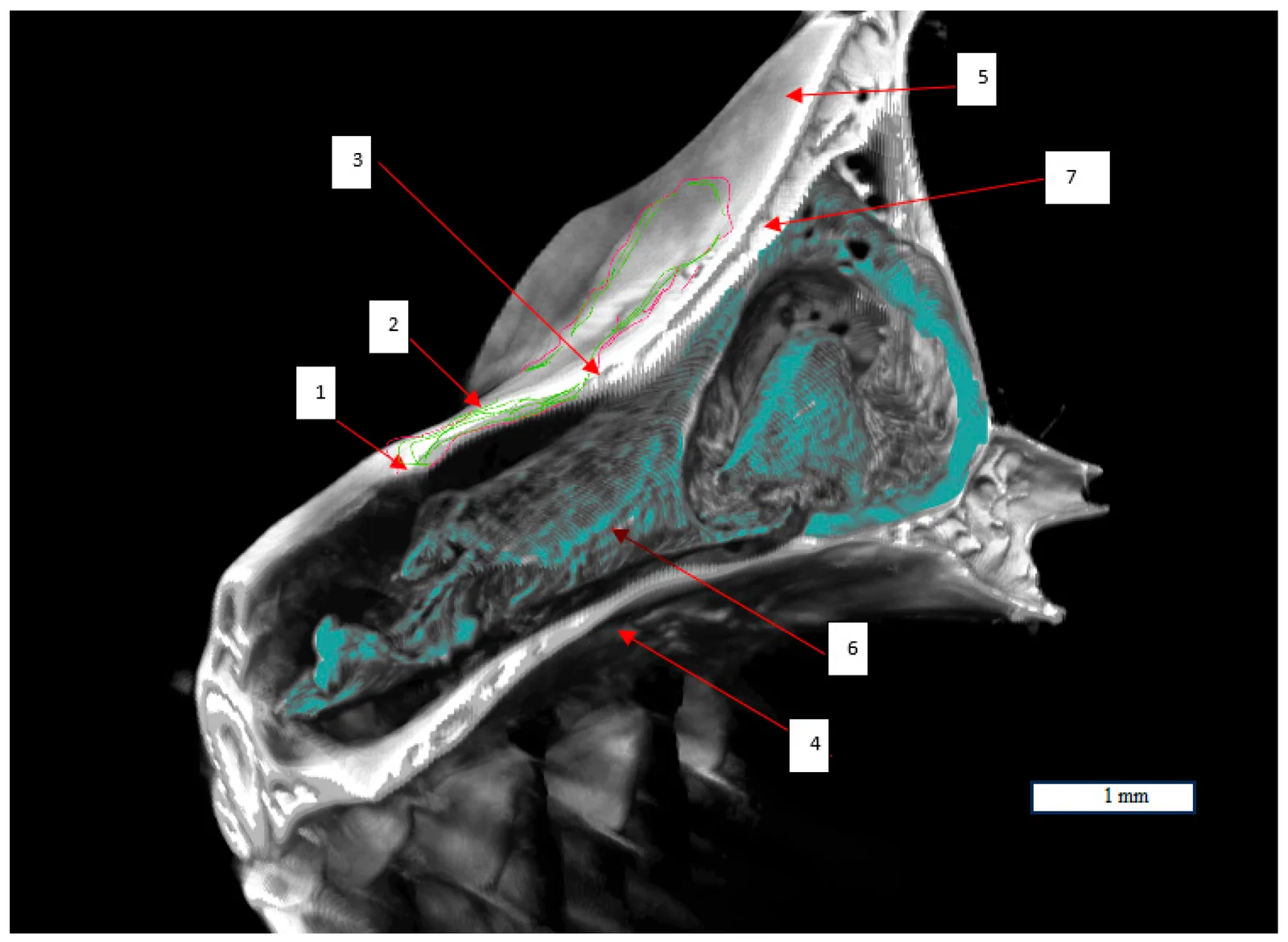
Oblique dorsolateral view of the nasofrontal region in *Plecotus auritus* (specimen no. 3). The figure illustrates the spatial relationships among the nasal bones, frontal bone, and the anterior portion of the ethmoidal region. Visible structures include the apex of the median complex (*vertex processus septalis*), the nasal bone (*os nasale*), the *internasal sagittal impression*, the palatine bone (*os palatinum*), the frontal bone (*os frontale*), the ethmoidal labyrinth (*pars labyrinthica ossis ethmoidalis*), and *processus internasalis ossis frontalis* (*nova structura*). The figure illustrates the frontal origin of *processus internasalis ossis frontalis* and its course between the paired nasal bones, where it occupies a central position within the nasofrontal region. Scale bar = 1 mm. Labels: 1. Vertex processus septalis; 2. Os nasale; 3. Internasal sagittal impression; 4. Os palatinum; 5. Os frontale; 6. Pars labyrinthica ossis ethmoidalis; 7. Processus internasalis ossis frontalis (nova structura).

Three-dimensional reconstruction revealed the presence of a previously undescribed frontal-derived osseous structure designated here as *processus internasalis ossis frontalis* (*nova structura*; Figure 2, label 4; Figure 3, label 7). This structure represents a rostral extension of the frontal bone (*os frontale*) and projects between the paired nasal bones along the median plane.

In an oblique dorsolateral view, *processus internasalis ossis frontalis* appears as an elongated, wedge-shaped element extending rostrally from the main body of the frontal bone (Figure 3, label 7). Its dorsal surface remains continuous with the dorsal surface of the frontal bone, whereas its lateral margins contact the medial surfaces of the paired nasal bones.

Rostrally, *processus internasalis ossis frontalis* reaches the region corresponding to the internasal sagittal impression (Figure 2, label 2; Figure 3, label 3). As a result, the caudal portions of the paired nasal bones do not contact each other directly but remain separated by this frontal-derived structure.

Figure 3 demonstrates the spatial relationships among *processus internasalis ossis frontalis*, the nasal bones (*ossa nasalia*), the frontal bone (*os frontale*), the ethmoidal labyrinth (*pars labyrinthica ossis ethmoidalis*), and the palatine bone (*os palatinum*). The structure occupies a central position within the roof of the nasal region and remains in direct topographical association with the median organization of this region.

*Processus internasalis ossis frontalis* was present in all examined specimens. Its position and spatial relationships were consistent throughout the analyzed material.

### 3.4. Relationship Between the Frontal Bone and the Osseous Nasal Septum

**Figure 4:**
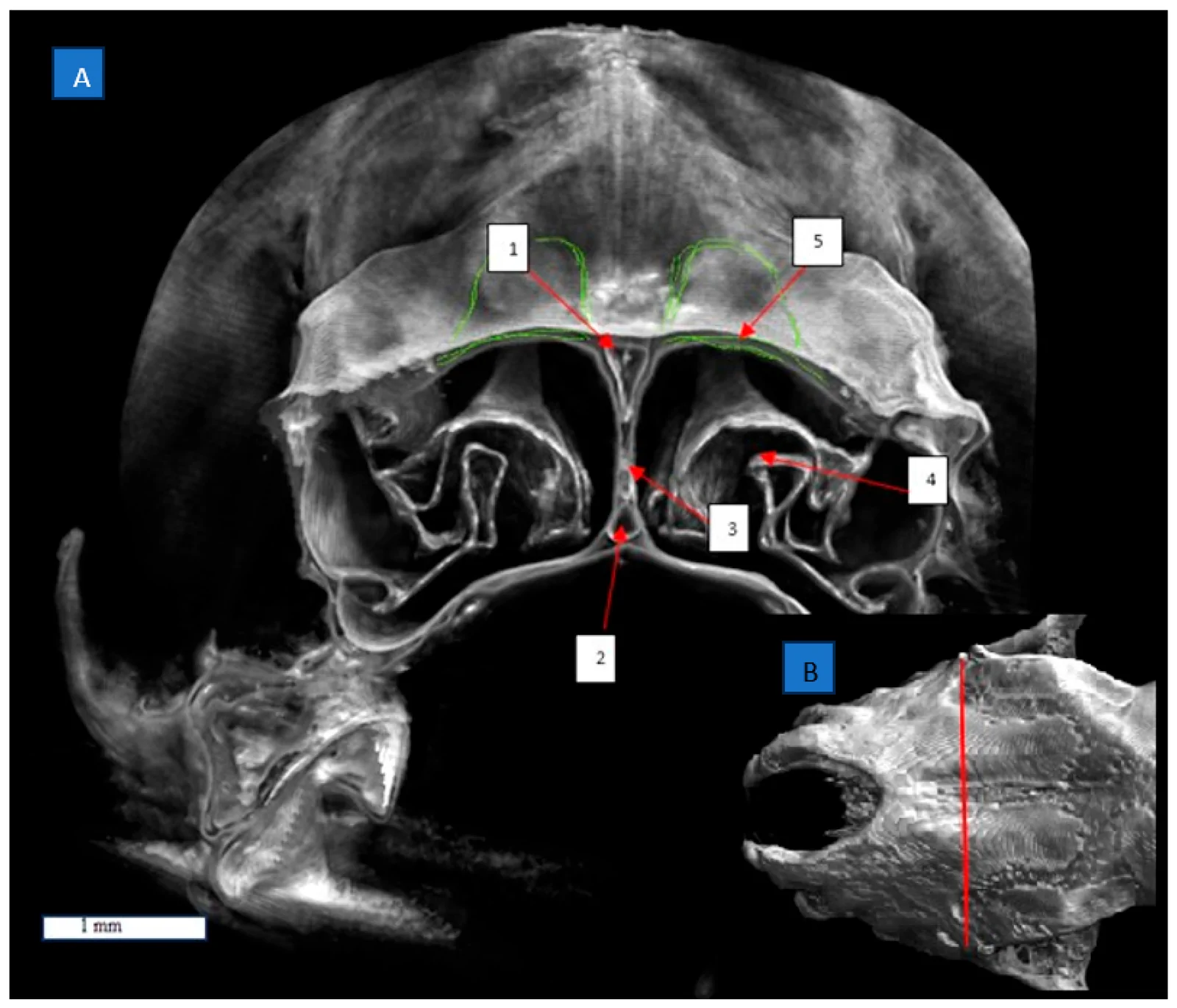
Transverse section through the nasofrontal region in *Plecotus auritus* (specimen no. 3). The figure illustrates the spatial relationships among *processus internasalis ossis frontalis* (*nova structura*), the osseous nasal septum, and the paired nasal bones. The visible structures include the rostral termination of *processus internasalis ossis frontalis*, the vomer (*vomer*), the osseous nasal septum (*septum nasi*), the ethmoid bone (*os ethmoidale*), and the nasal bone (*os nasale*). The arrangement of these structures demonstrates the direct topographical relationship between the frontal-derived process and the median axis of the osseous nasal septum. Panel (**B**) shows the location of the transverse section illustrated in panel (**A**). Scale bar = 1 mm. Labels: 1. Processus internasalis ossis frontalis (nova structura)—rostral termination; 2. Vomer; 3. Septum nasi; 4. Os ethmoidale; 5. Os nasale.

Structures incorporated into the model and documented in previous figures:

1. *Internasal sagittal impression*—Figure 1, label 6; Figure 2, label 2; Figure 3, label 3.
2. *Ala ossis nasalis* (*nova structura*)—Figure 1, label 8; Figure 2, label 5.
3. *Facies parasagittalis ossis nasalis* (*nova structura*)—Figure 2, label 6.
4. *Processus internasalis ossis frontalis* (*nova structura*)—Figure 1, label 9; Figure 2, label 4; Figure 3, label 7; Figure 4, label 1.
5. Relationship to the frontal bone (*os frontale*)—Figure 3, label 5.
6. Relationship to the osseous nasal septum (*septum nasi osseum*)—Figure 4, labels 2–3.

The organizational pattern observed in *Plecotus auritus* is characterized by the presence of *ala ossis nasalis* (*nova structura*), *facies parasagittalis ossis nasalis* (*nova structura*), *processus internasalis ossis frontalis* (*nova structura*), and the internasal sagittal impression.

Schematic model based on micro-computed tomography reconstructions and transverse sections presented in Figure 1, Figure 2, Figure 3 and Figure 4.

Analysis of the micro-CT sections revealed a close spatial relationship between *processus internasalis ossis frontalis* and the median osseous elements of the nasal cavity (Figure 3 and Figure 4).

In transverse section (Figure 4), the osseous nasal septum (*septum nasi osseum*; Figure 4, label 3) is visible along the median axis of the nasal cavity. Its ventral portion is formed by the vomer (*vomer*; Figure 4, label 2), whereas the laterally positioned ethmoidal structures are represented by the ethmoidal labyrinth (*pars labyrinthica ossis ethmoidalis*; Figure 4, label 4).

In the dorsal portion of the septal complex, the rostral termination of *processus internasalis ossis frontalis* is visible (Figure 4, label 1). This structure occupies a central position between the paired nasal bones (*ossa nasalia*; Figure 4, label 5) and remains in direct topographical continuity with the median axis of the osseous nasal septum.

The transverse section demonstrates that the caudal portions of the paired nasal bones do not form direct contact within the median plane. Instead, a frontal-derived element corresponding to *processus internasalis ossis frontalis* is interposed between them and occupies a position aligned with the median axis of the osseous nasal septum.

Observations obtained from transverse sections are consistent with the three-dimensional reconstructions presented in Figure 3, in which the frontal bone is seen extending rostrally between the paired nasal bones. In all examined specimens, the complex comprising *processus internasalis ossis frontalis*, *septum nasi osseum*, and the paired nasal bones exhibited the same spatial organization.

### 3.5. Modification of the Nasofrontal Articulation

Analysis of three-dimensional reconstructions and micro-CT sections (Figure 2, Figure 3 and Figure 4) demonstrated that the relationship between the paired nasal bones (*ossa nasalia*) and the frontal bone (*os frontale*) in *P. auritus* differs from the generalized mammalian pattern of nasofrontal organization.

In the examined material, the caudal portions of the paired nasal bones did not contact each other directly within the median plane. Instead, they were separated by *processus internasalis ossis frontalis*, a rostral extension of the frontal bone projecting between the paired nasal bones.

The presence of this structure resulted in separation of the caudal portions of the nasal bones and modified the organization of the region corresponding to the nasofrontal articulation. The paired nasal bones remained in direct contact only in the rostral portion of the nasal region, whereas caudally they were separated by *processus internasalis ossis frontalis*. This arrangement was consistently associated with the occurrence of the internasal sagittal impression, *ala ossis nasalis*, and *facies parasagittalis ossis nasalis*, all of which were present in every examined specimen.

**Figure 5:**
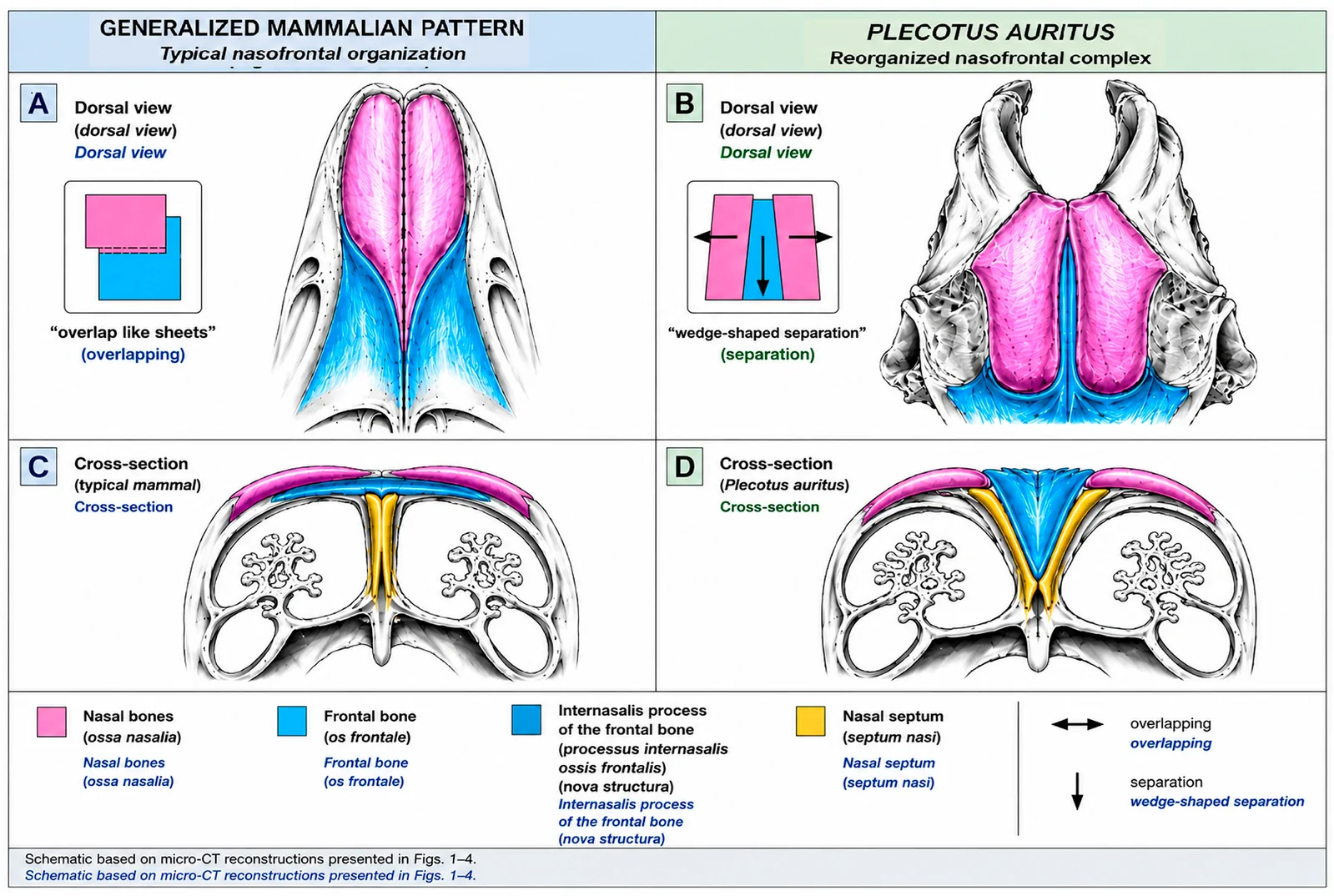
Comparative model of the generalized mammalian and *Plecotus auritus* patterns of nasofrontal organization. (**A**). Generalized mammalian pattern in dorsal view. The paired nasal bones (*ossa nasalia*) extend onto the frontal bone (*os frontale*), forming a direct nasofrontal contact. (**B**). Dorsal view of the pattern observed in *Plecotus auritus*. *Processus internasalis ossis frontalis* (*nova structura*) projects between the paired nasal bones, separating their caudal portions and modifying the architecture of the nasofrontal complex. (**C**). Generalized mammalian pattern in transverse section. The axis of the nasal septum is positioned between the paired nasal bones without the involvement of a frontal-derived median element. (**D**). Transverse section of the pattern observed in *Plecotus auritus*. *Processus internasalis ossis frontalis* occupies a position along the median axis of the osseous nasal septum (*septum nasi osseum*) and extends between the paired nasal bones.

The spatial relationships among these structures are summarized in the comparative model presented in Figure 5, which is based on observations derived from three-dimensional reconstructions and micro-CT sections.

### 3.6. Comparative Model of the Nasofrontal Complex

Figure 5 presents a comparative schematic model of the nasofrontal complex in a generalized mammalian condition and in *P. auritus*. The model was developed from observations derived from three-dimensional reconstructions and micro-CT sections presented in Figure 1, Figure 2, Figure 3 and Figure 4.

In the generalized mammalian pattern, the nasal bones (*ossa nasalia*) contact the frontal bone (*os frontale*) through a direct nasofrontal articulation. In dorsal view, the caudal portions of the nasal bones extend onto the rostral margin of the frontal bone, forming a continuous boundary between the nasal and frontal regions (Figure 5A). In transverse section, the axis of the nasal septum is positioned between the paired nasal bones without the involvement of a frontal-derived median element (Figure 5C).

In *P. auritus*, a different spatial arrangement was observed. In the caudal portion of the nasal region, *processus internasalis ossis frontalis* is present as a rostral extension of the frontal bone projecting between the paired nasal bones (Figure 5B). In transverse section, this structure occupies a position aligned with the median axis of the osseous nasal septum and extends between the paired nasal bones (Figure 5D).

In the model proposed for *P. auritus*, *processus internasalis ossis frontalis* occurs in association with the internasal sagittal impression, *ala ossis nasalis*, and *facies parasagittalis ossis nasalis*. All of these features were present in every examined specimen.

The schematic model presented in Figure 5 provides a synthetic summary of the spatial relationships documented in Figure 1, Figure 2, Figure 3 and Figure 4.

## 4. Discussion

### 4.1. Significance of Processus Internasalis Ossis Frontalis in Mammalian Anatomy

In the classical model of mammalian cranial organization, the paired nasal bones (*ossa nasalia*) form the roof of the nasal region, whereas the frontal bone (*os frontale*) defines its caudal boundary and contributes primarily to the anterior portion of the neurocranium [3,19]. Contact between these elements is typically established through the nasofrontal suture (*sutura frontonasalis*), without the presence of an intermediate skeletal element separating the paired nasal bones.

The present study revealed the presence of *processus internasalis ossis frontalis*, a rostral extension of the frontal bone projecting between the paired nasal bones. This structure was present in all examined specimens and exhibited consistent topographical relationships with both the nasal bones and the osseous nasal septum. These observations indicate that the structure does not represent an individual variation or a local morphological anomaly but rather a stable component of nasofrontal organization in *Plecotus auritus*.

To our knowledge, no structure corresponding morphologically and topographically to *processus internasalis ossis frontalis* has been described in the available osteological literature dealing with Mammalia or Chiroptera. Previous studies have focused primarily on general cranial morphology, cranial bone ontogeny, functional organization of the nasal region, or phylogenetic relationships [5,8,9], without considering the possibility of a median frontal extension separating the caudal portions of the paired nasal bones.

An important observation is that *processus internasalis ossis frontalis* does not occur as an isolated anatomical structure. In the examined material, it consistently co-occurred with the internasal sagittal impression, *ala ossis nasalis*, and *facies parasagittalis ossis nasalis*, together forming an integrated morphological complex occupying the median portion of the nasal roof. This consistent association suggests that these features should be interpreted as components of an integrated architectural arrangement rather than as independent modifications of individual cranial elements.

In light of the present findings, the role of the frontal bone in the organization of the nasal region of *P. auritus* appears to be more extensive than predicted by the generalized mammalian model. Rather than functioning solely as the caudal boundary of the nasal region, the frontal bone also contributes to the organization of its median axis through the development of *processus internasalis ossis frontalis*.

### 4.2. The Frontal Bone as an Organizing Element of the Nasofrontal Complex

In classical mammalian anatomy, the frontal bone (*os frontale*) is regarded primarily as a component of the cranial roof and as the caudal boundary of the nasal region [3,19]. Its involvement in the organization of the nasal portion of the skull is generally limited to forming the articulation with the nasal bones through the nasofrontal suture (*sutura frontonasalis*). Within this framework, the frontal bone functions principally as a boundary structure separating the nasal region from the neurocranial portion of the skull.

The results of the present study indicate that this relationship is modified in *P. auritus*. The presence of *processus internasalis ossis frontalis* means that the frontal bone does not terminate at the level of the nasofrontal articulation but instead extends rostrally between the paired nasal bones. Consequently, the frontal bone becomes an integral component of the median organization of the nasal region.

Particularly noteworthy is the relationship between *processus internasalis ossis frontalis* and the osseous nasal septum (*septum nasi osseum*). Micro-CT sections demonstrated that this process occupies a position aligned with the median axis of the nasal septum and remains in direct topographical association with it. This configuration suggests that the organization of the nasal region is not solely the result of the independent arrangement of individual skeletal elements but instead involves an integrated spatial relationship among the frontal bone, the nasal bones, and the median structures of the nasal cavity.

This interpretation is further supported by the consistent association of *processus internasalis ossis frontalis* with the internasal sagittal impression, *ala ossis nasalis*, and *facies parasagittalis ossis nasalis*. These structures do not occur as isolated modifications of individual bones but instead form a recurrent set of features occupying the median portion of the nasal roof. In this context, the frontal bone contributes not only to defining the caudal boundary of the nasal region but also to its internal spatial organization.

The most characteristic feature of this organization is the presence of a median frontal process separating the caudal portions of the paired nasal bones. As a consequence, the generalized pattern of direct contact between the nasal and frontal bones is not observed in the caudal median portion of the nasofrontal region. Instead, a frontal-derived element projects between the paired nasal bones. This arrangement is summarized in the comparative model presented in Figure 5, where the typical pattern of overlap between the nasal and frontal bones is contrasted with the wedge-shaped separation produced by *processus internasalis ossis frontalis*.

From a comparative anatomical perspective, these observations suggest that the contribution of the frontal bone to the organization of the nasal region may be more variable within Chiroptera than previously recognized from osteological studies [5,8]. However, verification of this hypothesis will require additional investigations involving representatives of other bat families and a broader comparative sample across Mammalia.

### 4.3. From Overlap to Separation: Two Models of Nasofrontal Organization

The classical model of mammalian cranial organization describes the relationship between the nasal bones (*ossa nasalia*) and the frontal bone (*os frontale*) as being based on direct surface contact. The nasal bones form the roof of the nasal region and contact the rostral margin of the frontal bone through the nasofrontal suture (*sutura frontonasalis*) while maintaining continuity within the median plane [3,19]. This arrangement may be interpreted as an overlap model, in which the boundary between the nasal and frontal regions is established through direct contact between adjacent skeletal elements.

The observations obtained from *P. auritus* indicate a different pattern of organization. The presence of *processus internasalis ossis frontalis* means that the frontal bone does not terminate at the boundary of the nasal region but instead projects between the paired nasal bones. As a consequence, the caudal portions of the nasal bones are separated by a frontal-derived element, and the classical pattern of nasofrontal articulation is modified.

The difference between the two organizational patterns is not limited to the presence of a single osseous structure. *Processus internasalis ossis frontalis* consistently co-occurs with the internasal sagittal impression, *ala ossis nasalis*, *facies parasagittalis ossis nasalis*, and the axis of the osseous nasal septum, forming an integrated anatomical complex occupying the median portion of the nasal region. This configuration is consistent with the concept of morphological integration, according to which modification of one anatomical component may be associated with coordinated reorganization of a broader structural complex [20,21].

From a morphological perspective, the arrangement observed in *P. auritus* may be described as a separation model, in which the frontal bone contributes to the organization of the median axis of the nasal region through the development of *processus internasalis ossis frontalis*. In contrast to the classical overlap model, where the boundary between the nasal and frontal regions is established through direct contact between adjacent skeletal elements, the observed pattern is characterized by spatial interposition of a frontal-derived structure between the paired nasal bones.

The model presented in Figure 5 synthesizes these differences and emphasizes that the observed reorganization involves the nasofrontal complex as a whole rather than a single osseous element.

It should be emphasized, however, that the present study is restricted to *P. auritus*. Whether the separation model represents a characteristic feature of the genus *Plecotus*, a broader pattern within Vespertilionidae, or a condition that also occurs in other chiropteran lineages remains unknown. Resolving this question will require further comparative investigations based on high-resolution micro-computed tomography.

### 4.4. Significance of Ala Ossis Nasalis and Facies Parasagittalis Ossis Nasalis

One of the most distinctive features of the nasal region in *P. auritus* is the presence of extensive lateral expansions of the nasal bones, designated here as *ala ossis nasalis*, together with associated parasagittal surfaces referred to as *facies parasagittalis ossis nasalis*. These structures have not been described in classical accounts of mammalian anatomy or in most available osteological studies of Chiroptera [3,5,19].

In the examined material, *ala ossis nasalis* forms a broad lateral expansion of the nasal bones, substantially increasing the width of the nasal roof. In turn, *facies parasagittalis ossis nasalis* constitutes an elongated surface extending parallel to the long axis of the nasal bone and remaining in close topographical association with the internasal sagittal impression.

Particularly noteworthy is the consistent association of both structures with *processus internasalis ossis frontalis*. The separation of the caudal portions of the nasal bones by a frontal-derived element results in a novel arrangement of spatial relationships within the roof of the nasal region. In this context, *ala ossis nasalis* and *facies parasagittalis ossis nasalis* may be interpreted as structures participating in the reorganization of the dorsal architecture of the nasal region.

Equally important is the spatial relationship between *facies parasagittalis ossis nasalis*, the internasal sagittal impression, and the axis defined by *processus internasalis ossis frontalis*. Together, these features form a continuous anatomical arrangement extending along the median plane, a configuration that is not present in the generalized model of mammalian nasal organization.

It may be hypothesized that the development of *ala ossis nasalis* is morphologically associated with the modification of the nasofrontal articulation. The lateral expansion of the nasal bones may represent a structural correlate of changes occurring within the median portion of the nasal roof. This interpretation remains hypothetical and requires verification using a broader comparative dataset.

An important observation is that both *ala ossis nasalis* and *facies parasagittalis ossis nasalis* were consistently present in all examined specimens. The recurrence of these features indicates that they do not represent expressions of individual morphological variation but instead constitute integral components of nasal-region organization in the studied species.

In a broader anatomical context, the presence of these structures further emphasizes that the reorganization of the nasofrontal complex in *P. auritus* cannot be attributed solely to a single frontal-derived process. Rather, it involves an entire set of co-occurring morphological features that collectively define a distinct pattern of nasal-region organization.

### 4.5. Relationship with the Osseous Nasal Septum

One of the most noteworthy observations obtained in the present study is the close association between *processus internasalis ossis frontalis* and the osseous nasal septum (*septum nasi osseum*). Analysis of micro-CT sections demonstrated that these structures maintain consistent topographical relationships and together define the median axis of the nasal region.

In the classical model of mammalian cranial organization, the nasal septum functions primarily as a partition separating the right and left nasal cavities. Its osseous component is formed mainly by the vomer (*vomer*) and elements of the ethmoid bone (*os ethmoidale*) [3,19]. The frontal bone is not generally considered a component directly involved in the organization of the osseous nasal septum or its median axis.

In *P. auritus*, a different arrangement is observed. *Processus internasalis ossis frontalis* occupies a position aligned with the axis of the nasal septum and represents its direct dorsal continuation. In transverse sections, this structure separates the caudal portions of the paired nasal bones and remains in close spatial association with the median elements of the nasal roof.

Of particular significance is the fact that this arrangement consistently co-occurs with the presence of the internasal sagittal impression. As a result, *processus internasalis ossis frontalis*, the internasal sagittal impression, and *septum nasi osseum* form a continuous set of anatomical features extending along the median axis of the nasal region. Such a configuration has not been described in the classical model of mammalian cranial organization.

The observed spatial continuity between the nasal septum and the internasal process of the frontal bone supports the interpretation of an integrated morphological arrangement involving the frontal bone, the nasal bones, and the median structures of the nasal cavity. This interpretation is consistent with the separation model proposed above, in which the frontal bone contributes to the organization of the axial component of the nasal region.

An additional important observation is that this arrangement was present in all examined specimens. This finding indicates that the relationship between *processus internasalis ossis frontalis* and *septum nasi osseum* represents a stable component of nasal-region organization in *P. auritus* rather than a consequence of individual morphological variation.

At the same time, the present results raise new questions concerning the significance of median cranial structures in the evolution of the chiropteran nasal region. Of particular interest is whether comparable relationships between the frontal bone, the osseous nasal septum, and the nasal bones occur in representatives of other bat families.

### 4.6. Evolutionary and Comparative Implications Within Chiroptera and Mammalia

The nasal region is among the most morphologically diverse components of the mammalian skull. Throughout mammalian evolution, it has undergone numerous modifications associated with the development of sensory systems, changes in feeding strategies, and reorganization of the respiratory apparatus [17,19]. Despite this diversity, the fundamental relationship between the nasal bones and the frontal bone has remained relatively conservative and is generally based on direct contact between these elements through the nasofrontal suture.

Within Chiroptera, morphological diversity is particularly evident in rostrum length, nasal-region proportions, organization of the nasal cavity, and structures associated with spatial orientation and echolocation [5,7,9]. Previous studies, however, have focused primarily on functional, developmental, and phylogenetic aspects, whereas detailed spatial relationships among the frontal bone, the nasal bones, and the median structures of the nasal region have received comparatively little attention.

The present findings indicate that *P. auritus* exhibits an organization of the nasofrontal complex that differs from the generalized mammalian pattern. The presence of *processus internasalis ossis frontalis*, *ala ossis nasalis*, and *facies parasagittalis ossis nasalis*, together with their consistent spatial relationships to the internasal sagittal impression and *septum nasi osseum*, supports the interpretation of an integrated morphological arrangement involving the median portion of the nasal region.

Particularly important is the fact that the observed reorganization does not involve a single bone or an isolated anatomical structure. Instead, it encompasses a coordinated set of co-occurring morphological features associated with the median axis of the nasal region. This pattern is consistent with the concept of morphological integration, according to which modifications affecting one anatomical component may be associated with coordinated transformations involving a broader anatomical complex [20,21].

In a broader evolutionary context, the present findings suggest that the median portion of the chiropteran nasal region may exhibit greater organizational diversity than previously recognized from osteological studies. The separation model observed in *P. auritus*, characterized by the presence of *processus internasalis ossis frontalis* projecting between the paired nasal bones, may represent a characteristic feature of the genus *Plecotus* or a broader organizational pattern within Vespertilionidae. Alternatively, it may constitute one of several organizational variants occurring within Chiroptera.

At present, it is not possible to determine the phylogenetic significance of the observed features. The lack of comparable high-resolution micro-computed tomography studies encompassing a broad taxonomic representation of bats precludes assessment of their distribution across chiropteran phylogeny. At the same time, the present study demonstrates that high-resolution micro-computed tomography can reveal previously unrecognized aspects of cranial organization, even within regions traditionally regarded as well understood.

Regardless of their phylogenetic interpretation, the observations presented here emphasize that the bat nasofrontal region may represent a valuable model for investigating morphological integration, developmental modularity, and the evolution of median cranial structures in mammals. Comparative studies involving representatives of different chiropteran families will be necessary to determine whether the organizational pattern described here represents a unique condition or forms part of a broader spectrum of morphological variation within the mammalian nasal region.

### 4.7. Limitations of the Study and Directions for Future Research

Several additional limitations should also be considered when interpreting the present findings.

First, the analysis was restricted to representatives of a single species. Although all examined skulls exhibited the same morphological features and similar spatial relationships among the structures described, it is currently not possible to determine whether the observed organizational pattern is characteristic exclusively of *P. auritus*, of the genus *Plecotus* as a whole, or whether it occurs more broadly within Vespertilionidae or other chiropteran lineages.

Second, the present study was morphological in nature and focused on the description of osseous structures and their spatial relationships. The available data do not allow a direct assessment of the functional significance of the observed features or of the developmental mechanisms responsible for their formation. Consequently, any interpretation regarding their biomechanical, sensory, or adaptive significance should be regarded as hypothetical pending independent verification.

Third, the analysis focused primarily on relationships among the frontal bone, the nasal bones, and the osseous nasal septum. Although the results indicate a close association among these elements within the median organization of the nasal region, a complete understanding of the observed arrangement will require consideration of additional components of the nasal skeleton, particularly the ethmoid bone (*os ethmoidale*) and its complex relationships with the structures forming the roof of the nasal cavity.

An important direction for future research will be comparative analyses involving additional species of the genus *Plecotus* as well as representatives of different chiropteran families. Such studies would make it possible to determine the extent of variation in the organizational pattern described here and to evaluate its potential phylogenetic significance.

Ontogenetic studies based on developmental material also appear particularly promising. Such investigations could help clarify the mechanisms underlying the formation of *processus internasalis ossis frontalis* and determine its relationship to the development of the nasal bones and the osseous nasal septum.

Another especially promising direction concerns the ethmoid bone (*os ethmoidale*) and its relationships with the median structures of the nasal region. The findings presented here suggest that the ethmoid bone may represent an important component of the organization of the nasofrontal complex and therefore warrants a separate, detailed investigation.

The present study demonstrates that high-resolution micro-computed tomography remains a powerful tool for revealing anatomical features that may be overlooked in conventional osteological analyses. The discovery of *processus internasalis ossis frontalis* and its associated structures indicates that even well-known elements of the mammalian skull can continue to provide new insights into the morphological organization and evolution of the nasal region.

## 5. Conclusions

High-resolution micro-computed tomography revealed a previously undescribed organization of the nasofrontal complex in *Plecotus auritus* (Linnaeus, 1758), differing from the generalized mammalian pattern of nasofrontal organization.

The most distinctive feature of this organization is the presence of *processus internasalis ossis frontalis* (*nova structura*), a median rostral extension of the frontal bone that separates the caudal portions of the paired nasal bones and modifies the architecture of the nasofrontal articulation.

This reorganization is associated with the occurrence of *ala ossis nasalis* (*nova structura*), *facies parasagittalis ossis nasalis* (*nova structura*), the internasal sagittal impression, and a close topographical relationship with the osseous nasal septum. Together, these features indicate that the observed pattern involves the nasofrontal complex as a whole rather than an isolated anatomical structure.

The observations presented here suggest that the nasofrontal organization observed in *P. auritus* may represent an alternative structural arrangement relative to the generalized mammalian condition. Whether this organization reflects a species-specific condition, a broader pattern within Chiroptera, or a more widespread mammalian characteristic requires verification through comparative studies.

The present findings demonstrate that well-known regions of the mammalian skull may still contain previously unrecognized information concerning anatomical organization and evolution while highlighting the value of high-resolution micro-computed tomography in comparative anatomical research.

## Data Availability

The datasets generated and/or analyzed during the current study are available from the corresponding author upon reasonable request.
